# Complement Factor I Mutation May Contribute to Development of Thrombotic Microangiopathy in Lupus Nephritis

**DOI:** 10.3389/fmed.2020.621609

**Published:** 2021-02-05

**Authors:** Min-Hua Tseng, Wen-Lang Fan, Hsuan Liu, Chia-Yu Yang, Jhao-Jhuang Ding, Hwei-Jen Lee, Shih-Ming Huang, Shih-Hua Lin, Jing-Long Huang

**Affiliations:** ^1^Division of Nephrology, Department of Pediatrics, Chang Gung Memorial Hospital, Taoyuan, Taiwan; ^2^Department of Pediatrics, Xiamen Chang Gung Hospital, Ximen, China; ^3^Genomic Medicine Core Laboratory, Chang Gung Memorial Hospital, Taoyuan, Taiwan; ^4^College of Medicine, Graduate Institute of Biomedical Sciences, Chang Gung University, Taoyuan, Taiwan; ^5^Molecular Medicine Research Center, Chang Gung University, Taoyuan, Taiwan; ^6^Division of Colon and Rectal Surgery, Lin-Kou Medical Center, Chang Gung Memorial Hospital, Taoyuan, Taiwan; ^7^Department of Microbiology and Immunology, College of Medicine, Chang Gung University, Taoyuan, Taiwan; ^8^Department of Otolaryngology-Head and Neck Surgery, Chang Gung Memorial Hospital, Taoyuan, Taiwan; ^9^Department of Pediatrics, Tri-Service General Hospital, National Defense Medical Center, Taipei, Taiwan; ^10^Department of Biochemistry, National Defense Medical Center, Taipei, Taiwan; ^11^Division of Nephrology, Department of Medicine, Tri-Service General Hospital, National Defense Medical Center, Taipei, Taiwan; ^12^Division of Asthma, Allergy, and Rheumatology, Department of Pediatrics, Chang Gung Memorial Hospital, Taoyuan, Taiwan

**Keywords:** lupus nephritis, thrombotic microangiopathy, genetic background, complement factor H, complement factor I gene

## Abstract

**Objective:** Renal thrombotic microangiopathy (TMA) is associated with complement overactivation and poor outcome in patients with lupus nephritis (LN). The role of genetic makeup of complement system in these patients remains to be elucidated.

**Methods:** The clinical and laboratory characteristics of 100 patients with LN during 2010–2017 were retrospectively analyzed. LN patients with renal TMA and condition-matched LN patients without renal TMA were studied. Twenty normal subjects were also enrolled for comparison. Whole exome sequence followed by Sanger sequence was used in our study cohort.

**Results:** Eight patients with renal TMA and eight condition-matched patients were enrolled from 100 LN patients with mean age 11.2 ± 2.0 years. Compared with condition-matched LN patients without renal TMA, LN patients with renal TMA exhibited statistically higher serum urea. Although most patients with renal TMA responded to plasma exchange, they had significantly higher relapse rate of nephritis, lower remission rate, and higher risk of end-stage renal disease and mortality. Compared with patients without renal TMA and normal subjects, those with renal TMA had significantly lower serum complement factor H (CFH) and plasma ADAMTS13 activity. Molecular analysis of all 100 patients with LN uncovered that three patients with renal TMA harbored mutations, two missense and non-sense, on *CFI* and *CFHR2*. The non-sense mutation, E302X, on *CFI* may impair its interaction C3b/CFH complex by loss of the heavy chain of complement factor I on simulation model.

**Conclusion:** In addition to low serum CFH level and plasma ADAMTS13 activity, defects in genes responsible for complement regulatory proteins may contribute to the development of renal TMA in patients with LN.

## Introduction

Lupus nephritis (LN) is one of the severe complications of systemic lupus erythematosus (SLE), and renal vasculopathy has been reported to be a poor prognosis marker of LN (1–3). Renal thrombotic microangiopathy (TMA) is a rare but potentially severe complication of LN and is associated with an increased risk of end-stage renal disease and death (4–7). The pathogenesis of TMA in LN remains to be elucidated. Recent investigations demonstrated that overactivation of complement pathways caused by genetic defects of serum complement regulatory proteins results in development of TMA (8–10). Plasmapheresis, which provides functional complement regulatory proteins, is previously the mainstay treatment for complement dysregulation in TMA caused by the defects of genes responsible for complement regulation (8–11). Currently, complement inhibitor, anti-C5 monoclonal antibody, has been demonstrated to have benefit on renal outcome and survival rate of LN patients with TMA (12, 13).

There is a paucity of literature in providing a comprehensive genetic analysis of LN patients with TMA. Without a clear understanding of how the genetic background may contribute to the pathogenesis of TMA, specific therapeutic strategies cannot be developed. We conducted this study to investigate the clinical features, outcome, and genetic characteristics in LN patients with TMA.

## Materials and Methods

### Patients and Normal Controls

The study protocol was approved by the Ethics Committee on Human Studies at Chang Gung Memorial Hospital, in Taiwan, Republic of China (201701388A3). Informed consent was obtained from the patients, normal subjects, and their parents after a detailed description of the study. All patients with SLE that fulfilled the American College of Rheumatology revised criteria and nephritis diagnosed by histology were enrolled between 2010 and 2017 (14).

### Demographic, Clinical, and Laboratory Characteristics

Patients demographics (sex and age) and clinical manifestations including age at disease onset, family history, underlying disease other than SLE, blood pressure, Systemic Lupus Erythematosus Disease Activity Index (SLEDAI), and organ involvement were analyzed. Laboratory data including hemoglobin, platelet count, schistocyte on smear, Coombs test, serum creatinine, lactate dehydrogenase, amylase, lipase, complement 3 (C3), complement 4 (C4), CH50, ADAMTS 13 activity (Technozyme ADAMTS-13 ELISA kit, Vienna, Austria), urine analysis, and *Streptococcus pneumoniae* antigen, and stool culture for shigatoxin-producing *Escherichia coli* at diagnosis of LN and/or renal TMA were recorded.

### Renal Histology

Renal histology examined under light microscopy, immunofluorescence, and electron microscopy was recorded. Histological classifications of LN were determined according to the ISN/RPS system (15, 16). Renal TMA was defined by histopathological features of endothelial cell swelling, lumen narrowing and obliteration, and thrombi formation in the interlobular artery, arteriole, and glomerular capillary (17, 18).

### Treatments and Follow-Up Outcome

Treatment including methylprednisolone, prednisolone, cyclophosphamide, mycophenolate mofetil, cyclosporine, and plasma exchange for LN was recorded. The response to plasma exchange was defined as the normalization of platelet count and hemoglobin level, and at least a 25% reduction of serum creatinine after five sessions of plasma exchange (19). Clinical outcomes including remission of LN, relapse of nephritis, end-stage renal disease, and mortality were recorded. Complete remission was defined as serum creatinine less than the upper limit of age and sex and protein <0.5 g/d or <4 mg/m^2^/h, and partial remission was defined as one of either a return to normal serum creatinine or urine protein <0.5 g/d (20). Relapse of LN and TMA was defined as an episode of LN or TMA within 4 weeks after partial or complete remission (21). End-stage renal disease was defined as creatinine clearance <15 mL/min/1.73 m^2^ or initiation of renal replacement therapy.

### Determination of Complement Regulatory Proteins Levels

The sera obtained at diagnosis from eight LN patients with renal TMA, eight condition-matched LN patients without renal TMA, and 20 normal subjects were used for determination of levels of serum complement regulatory proteins. The levels of serum complement factor H (CFH) (Abnova), complement factor I (CFI) (LSBio), membrane cofactor protein (MCP), and C3d (Blue Gene Biotech) were measured using enzyme-linked immunosorbent assay (ELISA) according to the manufacturer's guidelines (22). The linear portion of the standard curve was subsequently used for the measurement of serum CFH, CFI, MCP, and C3d. All assays were run in duplicate, and when standard errors were >10%, samples were reanalyzed.

### Determination of Anti-CFH and Anti-ADAMTS13 Autoantibodies

The sera drawn at the onset of renal TMA were used for measurement of autoantibodies. The presence of autoantibodies against CFH and ADAMTS13 was determined by using CFH and ADAMTS13 IgG ELISA kits (Abnova, Taipei, Taiwan) according to the manufacturer's instructions.

### Molecular Analysis of Corresponding Genes by Whole-Exome Sequencing

We performed molecular analysis after the onset of TMA. Genomic DNA was isolated from peripheral venous blood sample. The genes involved in complement system were analyzed. We performed exome capture using the Agilent SureSelect Human All Exon Kit 58 m (v6) (Agilent Technologies) and massively parallel sequencing using the HiSeq 4000 platform (Illumina, San Diego, CA) to generate paired-end 150-bp reads from genomic DNA sequencing in Biotools (New Taipei City, Taiwan). Raw image analyses and base calling were performed using Illumina's Pipeline with default parameters. Sequence data were aligned to the reference human genome (hg38) using the Burrows-Wheeler Aligner, and duplicate reads were removed using Picard tools (23). We used the Genome Analysis ToolKit (GATK) to perform the realignment and variation (SNP and InDel) detection (24). Annovar was utilized to catalog the detected variations (25). Then, we filtered variations with a homopolymer length >6 (and synonymous substitutions) or that were common (>2%) in dbSNP150 (http://www.ncbi.nlm.nih.gov/projects/SNP/), HapMap, the 1000 Genomes Project (http://www.1000genomes.org), the Exome Aggregation Consortium database, and the Genome Aggregation Database (gnomAD, https://gnomad.broadinstitute.org). Integrated Genome Viewer was used to visualize the reads and compare polymorphisms between each sequenced individual. Direct Sanger sequencing was performed to verify the genetic variants detected by whole-exome sequencing (WES). Pathogenicity score was calculated using SIFT (26), PolyPhen2 (27), LRT (28), MutationTaster (29), FATHMM (30), M-CAP (31), CADD (32), and GERP (33). Cutoffs defining pathogenic or benign assertion are either not recommended or inferred arbitrarily. Direct Sanger sequencing was performed for patients with identified mutations and their parents to verify the genetic variants detected by WES.

### Simulation of the Mutation Models

The resolved structures of human CFI (PDB code:2XRC) were used as template to generate the S221C and E302X mutants using the Built Mutants protocol (Biovia Discovery Studio 2019). The geometries of the models were optimized using the algorithm of smart minimization in CHARMm force field. The ternary complex of FI, factor H, and complement C3b was generated by overlaying the structure of FI to the binary complex of C3b–factor H (PDB code: 2WII) followed by geometry minimization (34).

### Statistical Analysis

The data were analyzed by using Statistical Package for Social Sciences, version 24.0 (SPSS Inc., Chicago, IL). The continuous variables were compared by Mann-Whitney *U*-test, and categorical data were compared using the χ^2^-test or Fisher exact test. Statistical significance was defined as *p* < 0.05.

## Results

### Demographic and Clinical Manifestations of Patients With LN

**Figure 1:**
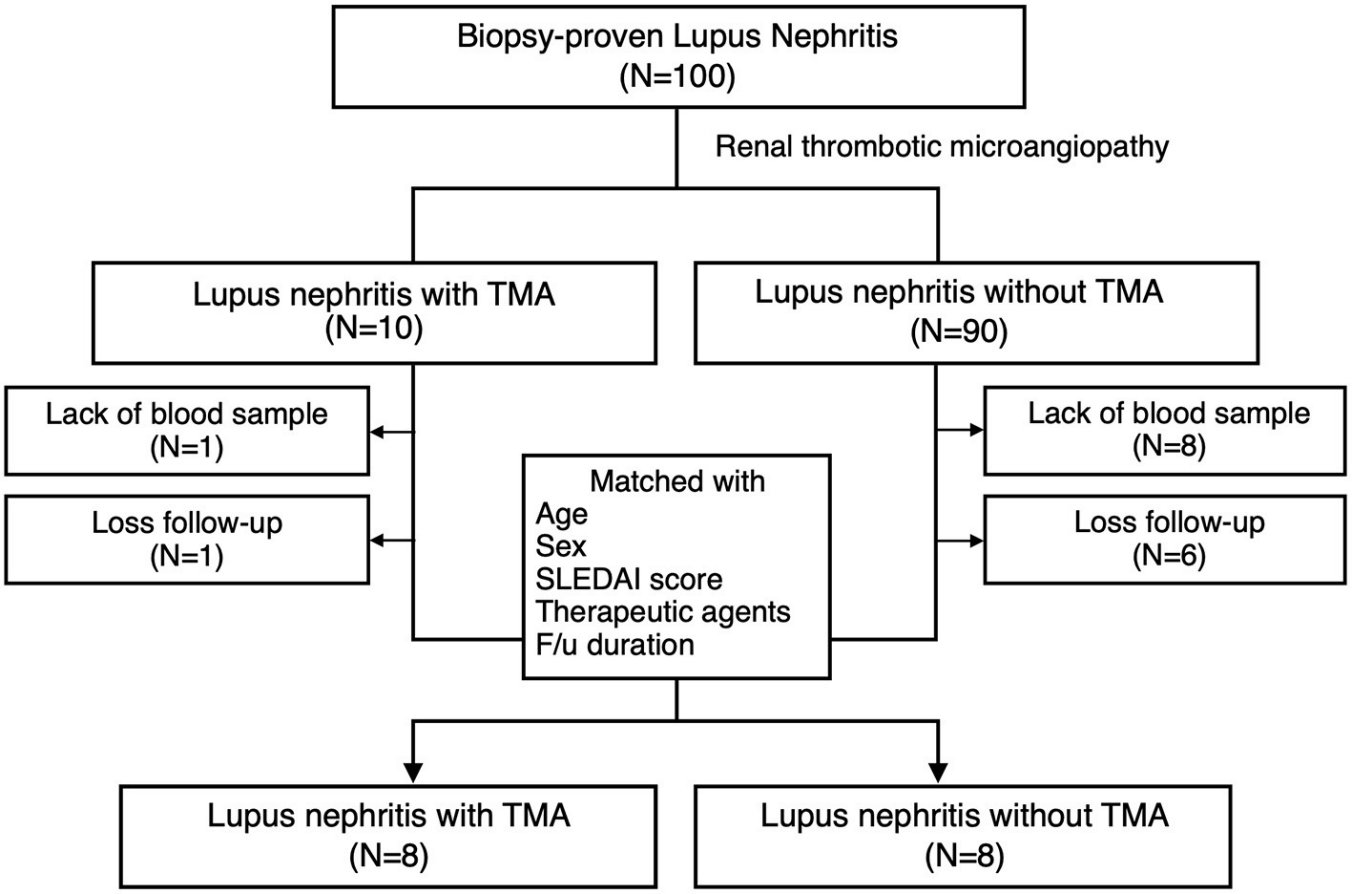
Patient enrollment. Inclusion criteria of lupus nephritis patients with and without thrombotic microangiopathy and the design of the study.

Ten LN patients with renal TMA and 90 LN without renal TMA were enrolled (Figure 1). Two LN patients with renal TMA and 14 LN patients without TMA were excluded because of the lack of DNA sample and loss to follow-up. Therefore, eight LN patients with renal TMA were analyzed. Another eight control LN patients matched for LN patients with TMA by age, sex, date of diagnosis, SLEDAI, histological severity of nephritis, medications, and duration of follow-up were recruited (Table 1). Five of LN patients (5/8, 62.5%) with renal TMA had acute kidney injury at presentation (Table 2). All LN patients with renal TMA did not have features of extrarenal TMA, except for patient three who died of intracranial hemorrhage.

**Table 1:** Clinical characteristics of lupus nephritis patients with and without thrombotic microangiopathy.

	**LN with TMA (*n* = 8)**	**LN without TMA (*n* = 8)**	***p*-value**
**Demography**			
Age	11.38 ± 5.66	15.5 ± 6.503	0.223
Sex (male/female)	1/7	1/7	1.0
Systolic pressure (mm Hg)	131.50 ± 21.35	128.13 ± 11.99	0.834
Diastolic pressure (mm Hg)	92.50 ± 17.30	86.38 ± 14.88	0.528
SLEDAI	16.25 ± 0.96	15.25 ± 0.84	0.446
**Laboratory**			
C3 (mg/dL)	43.74 ± 31.55	55.14 ± 38.16	0.600
C4 (mg/dL)	8.94 ± 7.53	7.37 ± 3.54	0.674
Anti-dsDNA (WHO units/mL)	422.66 ± 357.06	779.09 ± 768.24	0.344
Hemoglobin (g/dL)	8.96 ± 1.45	10.35 ± 2.22	0.172
Platelets (1,000/μL)	138.38 ± 102.46	259.38 ± 120.07	0.059
Creatinine (mg/dL)	1.64 ± 1.41	0.70 ± 0.30	0.115
BUN (mg/dL)	50.46 ± 27.25	19.86 ± 13.99	0.018
Albumin (g/dL)	2.86 ± 0.79	3.17 ± 0.72	0.451
**Renal histology**			
III	1	1	0.767
IV (segmental/global)	7 (1 IV-S, 6 IV-G)	7 (1 IV-S, 6 IV-G)	0.767
Thrombotic microangiopathy	8	0	0
**Treatment**			
Plasma exchange (%, no. of sessions)	8 (100%, 5.3 ± 0.6)	0 (0%)	<0.001
Methylprednisolone pulse (%)	8 (100%)	8 (100%)	1.000
Corticosteroid after MP^+^ (%)	8 (100%)	8 (100%)	1.000
Cyclophosphamide pulse (%)	6 (75%)	6 (75%)	1.000
Mycophenolate mofetil (%)	2 (25%)	2 (25%)	1.000
**Follow-up outcomes**			
Duration (month)	40.12 ± 13.6	41.25 ± 13.4	0.916
Response to plasma exchange (%)	7 (87.5%)	—	—
Remission of lupus nephritis^++^ (%)	4 (50.0%)	7 (87.5%)	0.045
Relapse of lupus nephritis	5 (62.5%)	1 (12.5%)	0.040
Relapse of thrombotic microangiopathy	2 (25%)	0	0.740
End-stage renal disease	3 (37.5%)	0 (0%)	0.045
Mortality	3* (37.5%)	0 (0%)	0.045

+ Methylprednisolone pulse therapy. ^++^Partial and complete remission rates. *Two sepsis and one intracranial hemorrhage.

**Table 2:** Clinical, laboratory, and histological features of individual with lupus nephritis and concomitant thrombotic microangiopathy.

	**1**	**2**	**3**	**4**	**5**	**6**	**7**	**8**
Age (years)	15	12	18	4	15	8	13	4
Gender	Male	Female	Female	Female	Female	Female	Female	Female
Blood pressure (mm Hg)	146/86	128/83	136/105	128/101	120/75	129/101	133/77	105/63
C3 (90–120 mg/dL)	48.2	18.5	25.7	104	13	73.6	45.8	21.1
C4 (10–20 mg/dL)	6.46	3.27	8.23	25.7	2.5	13.3	6.17	5.87
CH50 (63–145 CAE units)	154	188	158	24	46	66	28	36
C3d (4.7–7.7 μg/mL)	2.9	1.0	2.3	3.3	5.0	3.0	2.8	6.2
Anti-dsDNA (<109 units/mL)	1,097	406.5	544.6	49.5	343	93.8	132.9	714
Platelet (150–400 × 10^3^/μL)	201	55	24	314	61	90	123	239
Hgb (11–13 g/dL)	10.7	9.5	8.7	9.4	11	7.4	7.8	7.2
Creatinine (0.6–0.9 mg/dL)	1.55	2.42	4.75	0.6	0.76	1.33	1.35	0.38
Anticardiolipin IgG (<10 GPL-U/mL)	3.6	2.9	9.9	54.6	1.2	0.5	4.2	3.9
Anticardiolipin IgM (<10 MPL-U/mL)	5.0	5.5	8.2	10.4	2.3	5.7	2.4	9.1
ADAMTS-13 (%)	82.8	40.3	80.2	53.0	76.8	49.0	43.5	65.0
CFH (330–680 μg/mL)	289.922	190.24	186.76	159.75	173.70	158.01	171.09	205.90
CFI (40–80 μg/mL)	11.83	4.36	8.47	20.26	6.51	16.50	17.21	9.37
CD46 (3.7–10.9 ng/mL)	34.2	33.5	35.3	28.5	32.5	23.8	33.5	31.6
Renal histology	TMA	TMA	TMA	TMA	TMA	TMA	TMA	TMA
Genetic defect	*CFI* S221C	*CFI* E302X	*CFHR2* N157I	—	—	—	—	—

### Laboratory Characteristics of LN Patients With and Without Renal TMA at Diagnosis

Most LN patients with renal TMA manifested the triad of TMA (anemia, thrombocytopenia, and acute kidney injury). As shown in Table 1, LN patients with renal TMA had relatively lower platelet count and significantly higher serum urea levels than those without renal TMA. All LN patients with renal TMA had normal serum vitamin B_12_ levels with absence of anticardiolipin antibody (except patient 4), urine pneumococcus antigen, shigatoxin-producing *E. coli* on stool, ADAMTS 13 activity, or medical history suggestive of drug-associated TMA. Therefore, all LN patients with TMA are hemolytic uremic syndrome (HUS); 4 of them are secondary to SLE, and one is HUS-associated antiphospholipid syndrome.

### Treatment and Outcome

There were no significant differences in pharmacotherapy including steroids, alkylating agent, and immunosuppressant between LN patients with and without TMA. All LN patients with TMA, as shown in Table 2, were treated with plasma exchange. Six were initiated within 24 h and two within 48 h from onset of disease. One and a half times estimated plasma volume was processed in each session of plasma exchange. Seven patients underwent five sessions of plasma exchange every other day, and one received seven sessions according to the clinical condition. As shown in Table 1, seven of eight LN patients with TMA (87.5%) responded to plasma therapy. Four and seven patients with and without TMA reached partial or complete remission of LN, respectively (*p* = 0.045).

### Follow-Up Outcome

LN patients with and without TMA were followed for a mean of 40.1 and 41.2 months, respectively. As shown in Table 1, relapse was more commonly in LN patients with TMA compared to those without TMA (5 vs. 1, *p* = 0.045). Among LN patients with renal TMA, two had recurrent TMA during follow-up. Three LN patients (patients one, two, and three) with TMA developed end-stage renal disease during follow-up (*p* = 0.045). Of note, patients two and five died of sepsis, and patient three died of intracranial hemorrhage (Table 1).

### Complement Activation in LN Patients With and Without TMA

**Figure 2:**
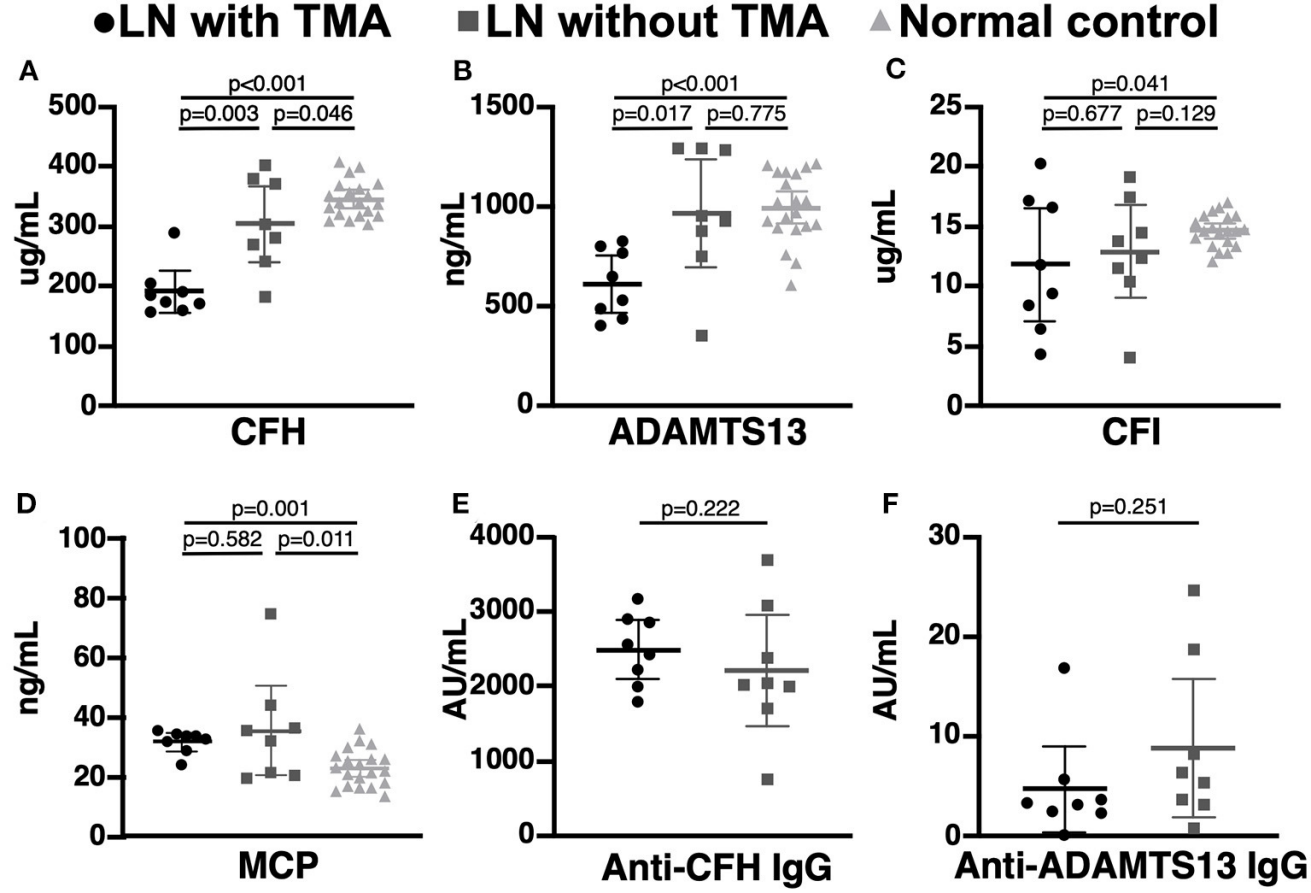
Complement and coagulation studies in normal subjects, lupus nephritis patients with and without renal thrombotic microangiopathy. **(A,B)** The serum CFH and ADAMTS13 levels were significant lower in lupus nephritis (LN) patients with thrombotic microangiopathy (TMA) than those without TMA and normal subjects. **(C,D)** LN patients with TMA have statistically lower CFI and MCP levels than normal subjects. **(E,F)** There was no significant difference in serum anti-CFH and anti-ADAMTS13 antibodies between LN patients with and without TMA.

Compared to LN patients without renal TMA and normal subjects, those with renal TMA had statistically lower serum CFH and ADAMTS13 levels. LN patients with renal TMA also had significantly lower serum CFI and higher MCP than normal subjects. Compared to normal subjects, LN patients without renal TMA had statistically lower serum CFH and higher MCP (Figure 2). There were no significant differences in anti-CFH and anti-ADAMTS13 antibody levels between those with and without renal TMA. The mean level of C3d in TMA patients with identified mutations (patients one, two, and three) and those without mutation are 2.07 and 4.06 μg/mL (*p* = 0.086), respectively.

### Mutations of Genes Corresponding to Complement Activation

**Figure 3:**
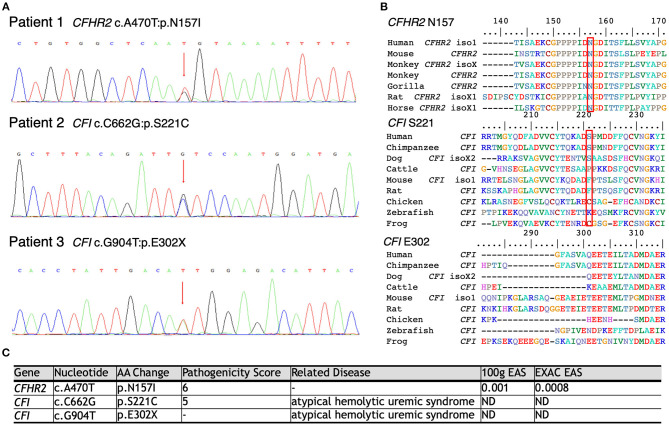
Schematic model of the CFHR2 and CFI proteins and genes. **(A)** Genetic defects of complement genes in lupus patients with renal thrombotic microangiopathy. **(B)** The N157 of *CFHR2* and *S221* of CFI are highly conserved amino acids. **(C)** High pathogenicity score of *CFHR2* N157I and of CFI *S221C*.

As shown in Figure 3, WES was conducted in all 100 LN patients. Sanger sequencing was performed for validation of mutations identified by WES. None of the LN patients without renal TMA had a defect in genes encoding complement regulatory proteins. On the other hand, three LN patients with renal TMA harbor non-synonymous mutations: two missense and one non-sense on *CFI* and *CFHR2* genes (p.S221C and E302X on *CFI* and p.N157I on *CFHR2*). Of note, two of these were novel mutations. These three variants were not detected in 200 healthy subjects, and the pathogenicity scores of *CFI* S221C and *CFHR2* N157I were 5 and 6, respectively. The N157 on *CFHR2* and S221 on *CFI* are highly conserved amino acid (Figure 3B). Both TMA patients with *CFI* mutations had relatively lower serum CFI levels than other TMA and non-TMA patients (*p* = 0.124 and 0.073, respectively). Of note, patients 2 and 3 who harbored *CFI* mutations died of sepsis and intracranial hemorrhage, respectively. Patient one who had *CFHR2* mutation progressed to end-stage renal disease during follow-up.

### Simulation of the Mutation Models

**Figure 4:**
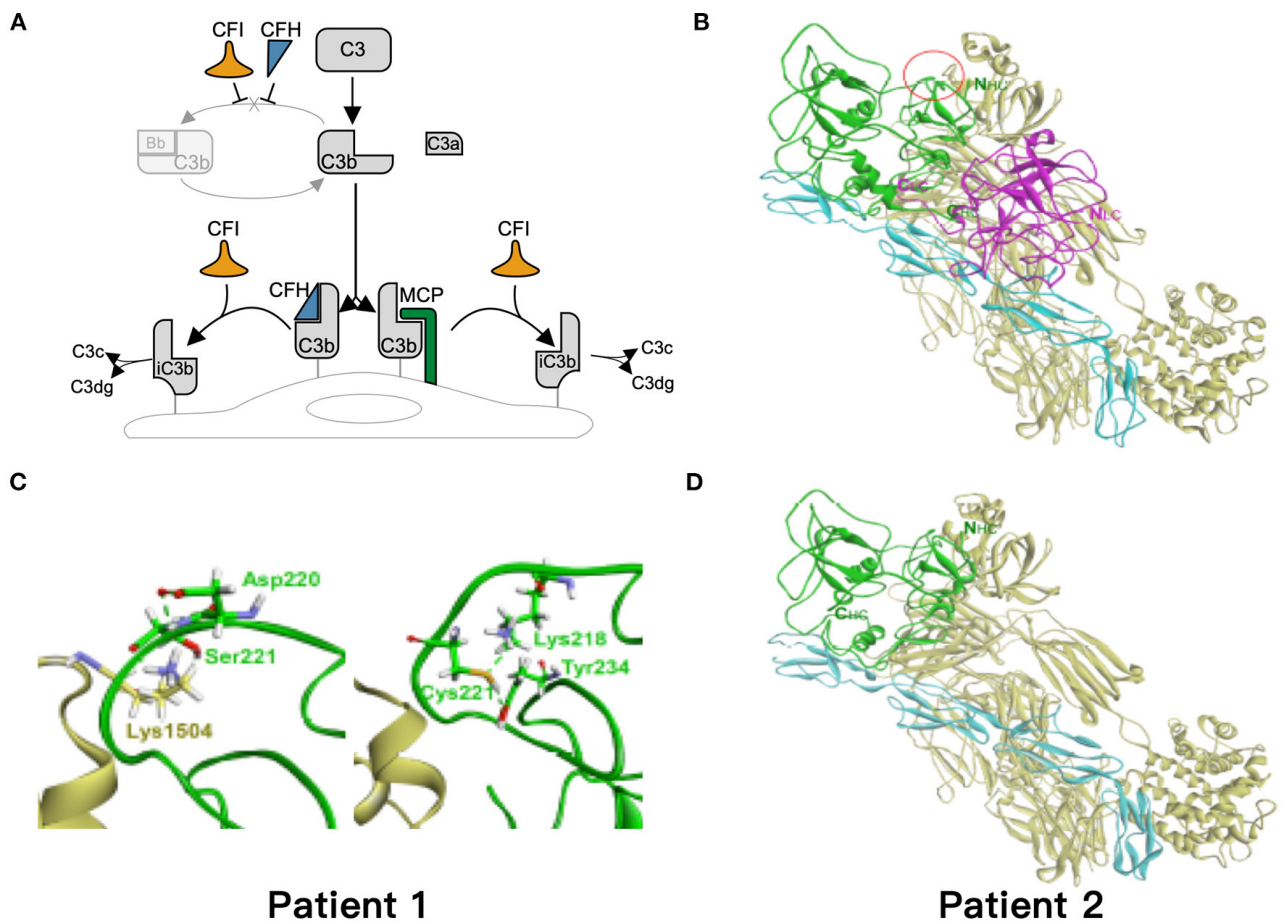
Simulation of the mutant models. **(A)** Regulation of the alternative complement pathway. CFI is responsible for the proteolytic inactivation of C3b to inactive C3b (iC3b) and irreversibly preventing reassembly of the C3 convertase. CFH and MCP acts as cofactors for CFI in the cleavage of C3b to iC3b. **(B)** The models of ternary complex of factor I (FI), factor H, and C3b. The proteins are presented as ribbon model and colored green and magenta for the heavy and light chain of FI, respectively, and cyan and yellow for factor H and C3b, respectively. **(C)** The red cycle represents the site for Ser221 in wild type and Cys221 in mutant FI, respectively. **(D)** The E302X mutant FI in complex with factor H and C3b. The hydrogen bond is shown as dashed green line.

Based on the critical roles of complement regulatory proteins in regulation of complement activation (Figure 4A), we performed simulation models to elucidate the possible impairment of interaction between C3b and CFI S221C and E302X. The CFI S221C mutant did not demonstrate apparent binding energy change with C3b/CFH complex, even though it had distinctive local interaction network with C3b. The red cycle represents the site for Ser221 in wild-type (Figure 4B) and Cys221 in mutant CFI (Figure 4C). The E302X mutant lost the heavy chain of CFI to complex with C3b/CFH (Figure 4D).

## Discussion

Within a large pediatric lupus cohort study in Taiwan, we investigated the role of gene mutations responsible for complete activation on clinical outcomes in LN patients with renal TMA. LN patients with renal TMA had more acute kidney injury and worse clinical outcome including high treatment failure, relapse of nephritis, end-stage renal disease, and higher mortality than those without renal TMA. Three different mutations involving the complement regulation genes were uncovered in three LN patients with TMA. The genetic background involving the complement regulation proteins, CFI and CFHR2, may play a role in the development of TMA in patients with LN. To the best of our knowledge, this is the first study to dissect the genetic background of pediatric LN patients with renal TMA.

In normal regulation of complement activation, CFI is responsible for the proteolytic inactivation of C3b to inactive C3b. CFH, CFHR, and MCP act as cofactors for CFI in the cleavage of C3b to iC3b. Three patients with LN combined with renal TMA have mutation in *CFI* and *CFHR2* genes responsible for complement regulation; this is consistent with previous findings in adult lupus patients (12, 13). Two heterozygous genetic variants, S221C and E302X on CFI, were identified in two LN patients with TMA. These two patients had decreased serum CFI levels. In addition, the amino acid, S221, is highly conserved, and the E302X mutant lost the heavy chain part of CFI to C3b-CFH complex according to the simulation models. Based on these findings, these two genetic variants may reduce or affect the interaction between CFI and C3b and thereby impairment of inactivation C3b. Patient 1 had a novel mutation, N157I on *CFHR2*. This amino acid, N157, is also highly conserved. Eberhardt et al. (35) identified CFHR2 as an alternative complement pathway regulator that inhibits C3 convertase and terminal pathway assembly by its action with CFH. In addition, da Holanda et al. (36) identified heterozygous deletion of *CFHR1*-*CFHR3* in case of LN with renal TMA. Therefore, the genetic abnormality of *CFHR2* may affect the interaction of CFH and C3b and then impairment of inactivation C3b. A functional study is still warranted to confirm the pathogenicity of these novel mutants on *CFI* and *CFHR2*. These variants are possibly pathogenic because these variants had extremely low allele frequency and were not identified in 200 healthy subjects. However, further functional assays are needed to prove that the novel mutations are indeed deleterious in patients with TMA. In summary, we propose that genetic background involving complement regulation proteins may shed light on the pathophysiology in the development of TMA in patients with LN.

In this study, we also found that LN patients with renal TMA had significantly lower serum CFH than those without renal TMA and normal subjects. Previous study has shown that low CFH may be one mechanism by which patients with LN develop TMA (8, 37). In addition, we also demonstrated that the serum activity of ADAMTS13 in LN patients with renal TMA were lower than that of LN patients without TMA. The principle function of ADAMTS 13 is cleavage of von Willebrand factor, whereby deficient or dysfunction of ADAMTS13 could result in vascular thrombi and TMA (38). In line with our findings, previous studies have demonstrated the utilization of serum ADAMTS13 level as a thrombotic marker of SLE (39, 40). Recent literature also showed that ADAMTS13 functions as a regulator of the complement system (41). Therefore, patients with lower ADAMTS13 activity may have a predilection in developing TMA via direct impairment of complement regulation and/or indirect promotion of vascular thrombi formation.

In our study, LN patients with renal TMA had lower remission rate and higher relapse rate of nephritis and were more likely to develop end-stage renal disease or die than those without renal TMA. In line with our findings, prior studies also showed that renal TMA is a strong risk factor for poor renal outcome and death (7, 8, 42). Among three TMA patients with mutations in genes involving the complement pathway (patients one, two, and three), two died of intracranial hemorrhage and sepsis, and the other developed end-stage renal disease. This suggests that the overall prognosis is poorer in LN patients with renal TMA, especially in those who may harbor mutations affecting the complement regulatory system. Although most LN patients with renal TMA responded to plasma exchange therapy, the long-term outcome remained poor. We speculate that there are two reasons to this finding. One is that plasma exchange may correct the low serum CFH, CFI, and ADAMTS13 levels only temporarily, but complement activation may reoccur or continue asymptomatically after treatment. In fact, this speculation is in accordance with the experiences from prior studies in patients with atypical hemolytic uremic syndrome, a type of TMA caused by complement overactivation. In the same regard, although these patients responded to plasma exchange therapy, their 1-year mortality and end-stage renal disease rates were as high as 60% (43). Second, plasma exchange may have been insufficient in providing adequate functional factors such as CFH, CFI, and ADAMTS13 in LN patients with renal TMA. From a therapeutic standpoint, a phase I study demonstrated therapeutic efficacy of complement inhibitor in patients with SLE, providing evidence in the link between complement dysregulation and disease. This was followed by studies that showed treatment success using eculizumab, a humanized anti-C5 monoclonal antibody, in an SLE patient with concomitant refractory TMA (12, 13, 44–46) and in LN patients with evidence of complement overactivation on kidney by finding of C9 deposition (46). These findings highlight the notion that complement blockade may have therapeutic benefit in LN with complement dysregulation or/and TMA. Given the success of these singular case reports, a randomized controlled trial to evaluate the potential therapy of anti-C5 monoclonal antibody in LN patients with renal TMA is warranted.

The current study has some limitations. First, a small patient size limits our ability to dissect the exact incidence of genetic defects of patients with renal TMA. However, we did identify defects in genes that are responsible for complement regulatory system, which could play an important role in the development of TMA in patients with LN. Second, we were not able to evaluate the therapeutic benefits of additional sessions of plasma exchange or anti-C5 therapy. Third, because of the nature of a retrospective study, only stored serum were available, and therefore plasma C3c, C3d, and sC5b-9 levels were not possibly measured.

In conclusion, renal TMA is a risk factor for poor outcomes in patients with LN. Defects on genes responsible for complement regulation as well as low serum ADAMTS13 and CFH levels may play an important role in the development of renal TMA in patients with LN.

## Data Availability Statement

The datasets presented in this study can be found in online repositories. The names of the repository/repositories and accession number(s) can be found in the article/supplementary material.

## Ethics Statement

The studies involving human participants were reviewed and approved by Ethics Committee on Human Studies at Chang Gung Memorial Hospital, in Taiwan, R.O.C. (201701388A3). Written informed consent to participate in this study was provided by the participants' legal guardian/next of kin.

## Author Contributions

M-HT, W-LF, HL, and C-YY substantially contributed to study conception and design, acquisition of data, and analysis and interpretation of data. J-JD, H-JL, S-HL, and S-MH substantially contributed to acquisition of data, and analysis and interpretation of data. M-HT and J-LH substantially contributed to study conception and design. All the authors revised the paper and approved the final version of the article to be published.

## Conflict of Interest

The authors declare that the research was conducted in the absence of any commercial or financial relationships that could be construed as a potential conflict of interest.
